# Meta-analysis accelerator: a comprehensive tool for statistical data conversion in systematic reviews with meta-analysis

**DOI:** 10.1186/s12874-024-02356-6

**Published:** 2024-10-18

**Authors:** Abdallah Abbas, Mahmoud Tarek Hefnawy, Ahmed Negida

**Affiliations:** 1 Faculty of Medicine, Al-Azhar University, Damietta, Egypt; 2 Faculty of Medicine, Zagazig University, Zagazig, Egypt; 3 Department of Neurology, Virginia Commonwealth University, Richmond, VA USA

**Keywords:** Meta-analysis, Statistical conversion, Data transformation, Research tool, Systematic review, Data analysis, Medical statistics, Meta converter, Meta-analysis accelerator

## Abstract

**Background:**

Systematic review with meta-analysis integrates findings from multiple studies, offering robust conclusions on treatment effects and guiding evidence-based medicine. However, the process is often hampered by challenges such as inconsistent data reporting, complex calculations, and time constraints. Researchers must convert various statistical measures into a common format, which can be error-prone and labor-intensive without the right tools.

**Implementation:**

Meta-Analysis Accelerator was developed to address these challenges. The tool offers 21 different statistical conversions, including median & interquartile range (IQR) to mean & standard deviation (SD), standard error of the mean (SEM) to SD, and confidence interval (CI) to SD for one and two groups, among others. It is designed with an intuitive interface, ensuring that users can navigate the tool easily and perform conversions accurately and efficiently. The website structure includes a home page, conversion page, request a conversion feature, about page, articles page, and privacy policy page. This comprehensive design supports the tool’s primary goal of simplifying the meta-analysis process.

**Results:**

Since its initial release in October 2023 as Meta Converter and subsequent renaming to Meta-Analysis Accelerator, the tool has gained widespread use globally. From March 2024 to May 2024, it received 12,236 visits from countries such as Egypt, France, Indonesia, and the USA, indicating its international appeal and utility. Approximately 46% of the visits were direct, reflecting its popularity and trust among users.

**Conclusions:**

Meta-Analysis Accelerator significantly enhances the efficiency and accuracy of meta-analysis of systematic reviews by providing a reliable platform for statistical data conversion. Its comprehensive variety of conversions, user-friendly interface, and continuous improvements make it an indispensable resource for researchers. The tool’s ability to streamline data transformation ensures that researchers can focus more on data interpretation and less on manual calculations, thus advancing the quality and ease of conducting systematic reviews and meta-analyses.

## Background

Meta-analysis systematically combines previous studies to provide precise estimates of effects, assess variability, and offer comprehensive insights into complex research findings [1]. By systematically pooling studies, researchers aim to address limitations in the size or scope of individual studies to obtain more reliable information about treatment effects [2]. The rigorous conduct of meta-analysis is crucial to ensuring its value in evidence-based medicine [3].

In contrast to a literature review, which compares and discusses study results, systematic reviews and meta-analyses synthesize data to reach new conclusions [4]. Unlike pooled data analysis, which uses individual subject data, systematic reviews and meta-analyses combine summarized results from multiple studies [5]. Although they are often perceived as simpler due to their lack of direct involvement with human or animal subjects, they require careful planning, much like any other research study [2]. They hold a prominent position in evidence-based medicine due to their high ranking in the clinical evidence hierarchy, owing to their strength and consistency [6].

Since the late 1970s, systematic reviews and meta-analyses have become integral to medical literature, with a substantial increase in publications. Their methodologies have advanced significantly, making them the most frequently used and cited forms of clinical research [7, 8].

Conducting the meta-analysis part of systematic reviews involves converting various statistical measures into a common format, which can be complex and time-consuming [9, 10]. Researchers face several challenges, including:

- **Inconsistent Reporting**: Studies often report data in different formats (e.g., means, medians, ranges, standard deviations (SDs)).
- **Complex Calculations**: Transforming data requires accurate calculations, which can be error-prone without the right tools.
- **Time Constraints**: Manual conversions are time-intensive, diverting focus from analysis and interpretation.

Meta-Analysis Accelerator [11] addresses these challenges by providing a comprehensive, user-friendly platform for statistical data conversion. This tool simplifies the conversion process, ensuring accuracy, efficiency, and consistency, allowing researchers to focus more on data interpretation and less on manual calculations.

In the landscape of tools available for conducting systematic reviews, our platform stands out as the first to specifically address the crucial need for data conversions, which are essential for facilitating the meta-analysis phase. While existing platforms like ‘Rayyan’ and ‘Covidence’ excel in screening and organizing studies, none offer dedicated functionalities for data conversions, which are pivotal for aggregating meta-analysis data. Other tools like ‘EPPI-Reviewer’ [12] and ‘Comprehensive Meta-Analysis’ [13], provide features like risk of bias assessment and comprehensive data analysis capabilities but still require researchers to preprocess data into a standardized format. Our platform uniquely bridges this gap by offering seamless data conversion tools, enabling researchers to efficiently prepare clean, homogeneous datasets for analysis.

Another available data conversion tool is the Review Manager (RevMan) Calculator [14], which has recently become available online but remains limited in terms of data conversion options. The RevMan Calculator is also available as an offline version for desktop use but is not cross-device accessible. It includes only the calculations for unadjusted odds ratio (OR), risk ratio (RR), hazard ratio (HR), and their logarithmic counterparts (log OR, log RR, log HR). In contrast, our platform currently provides 21 different data conversions, aiming to offer researchers all the required data conversions in one place. Additionally, our platform is easily accessible and entirely free of charge, enhancing its utility for researchers globally.

## Implementation

### Website structure

1. **Home Page or Landing Page**.
2. **Conversion Page**: Includes all the conversions.
3. **Request a Conversion**: Allows users to request a conversion not currently available.
4. **Contact Us**: Enables users to provide feedback and suggestions for improvements.
5. **About Page**: Explains our vision, mission, and reasons behind creating this tool.
6. **Articles Page**: To be added in the next update, explaining statistical concepts. Each conversion will link to an article explaining it.
7. **Privacy Policy Page**.

### How to use Meta-Analysis Accelerator

**Fig. 1:**
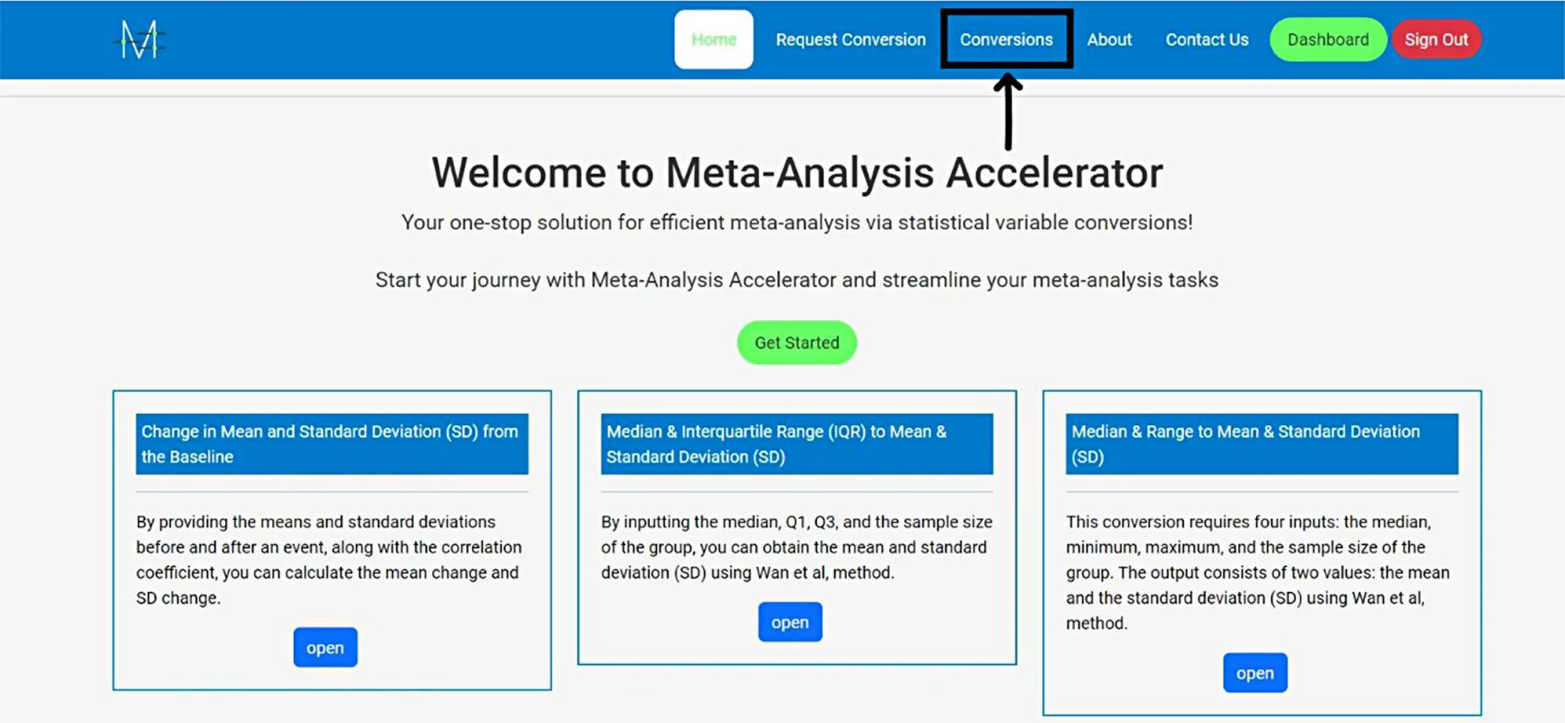
Go to the conversions page

**Fig. 2:**
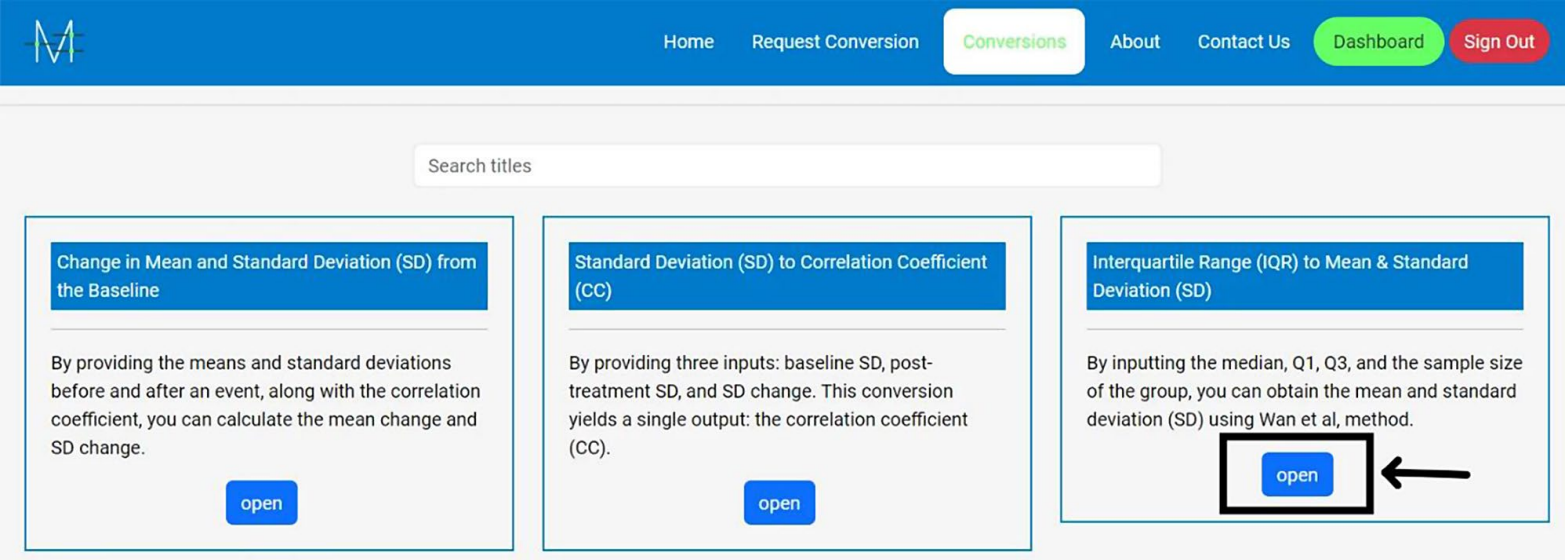
Select the conversion that matches your needs

**Fig. 3:**
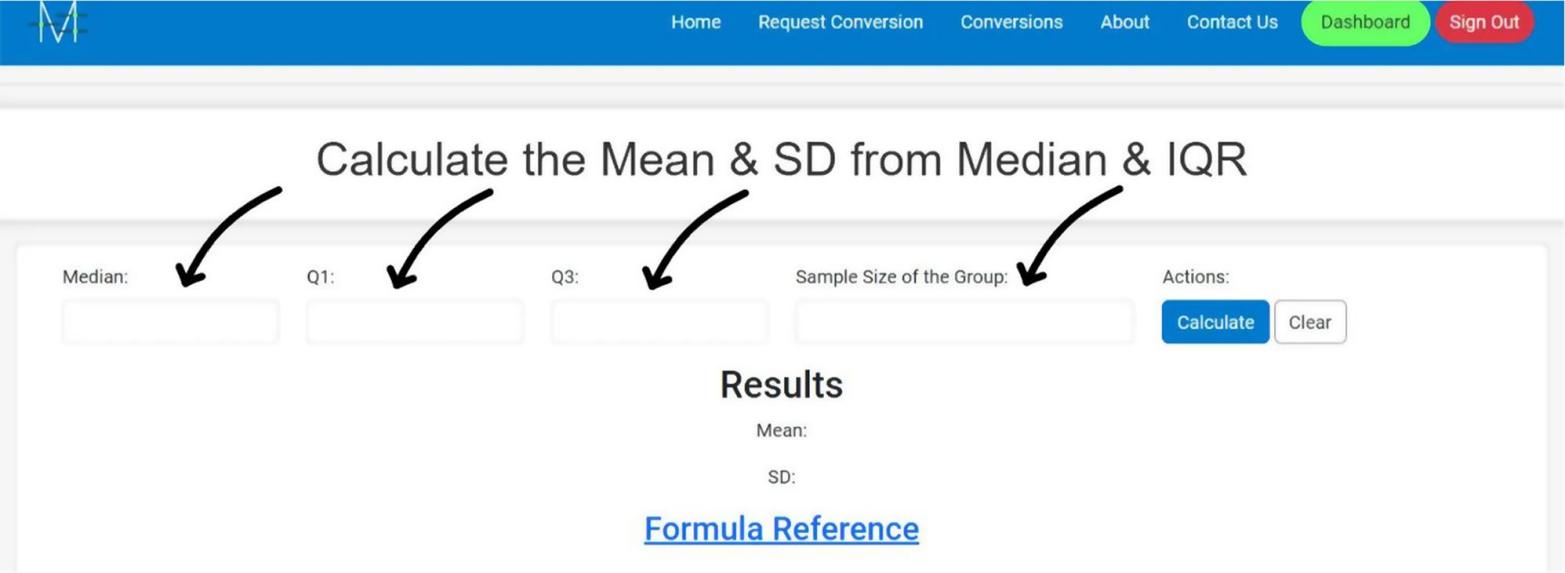
Enter the data

**Fig. 4:**
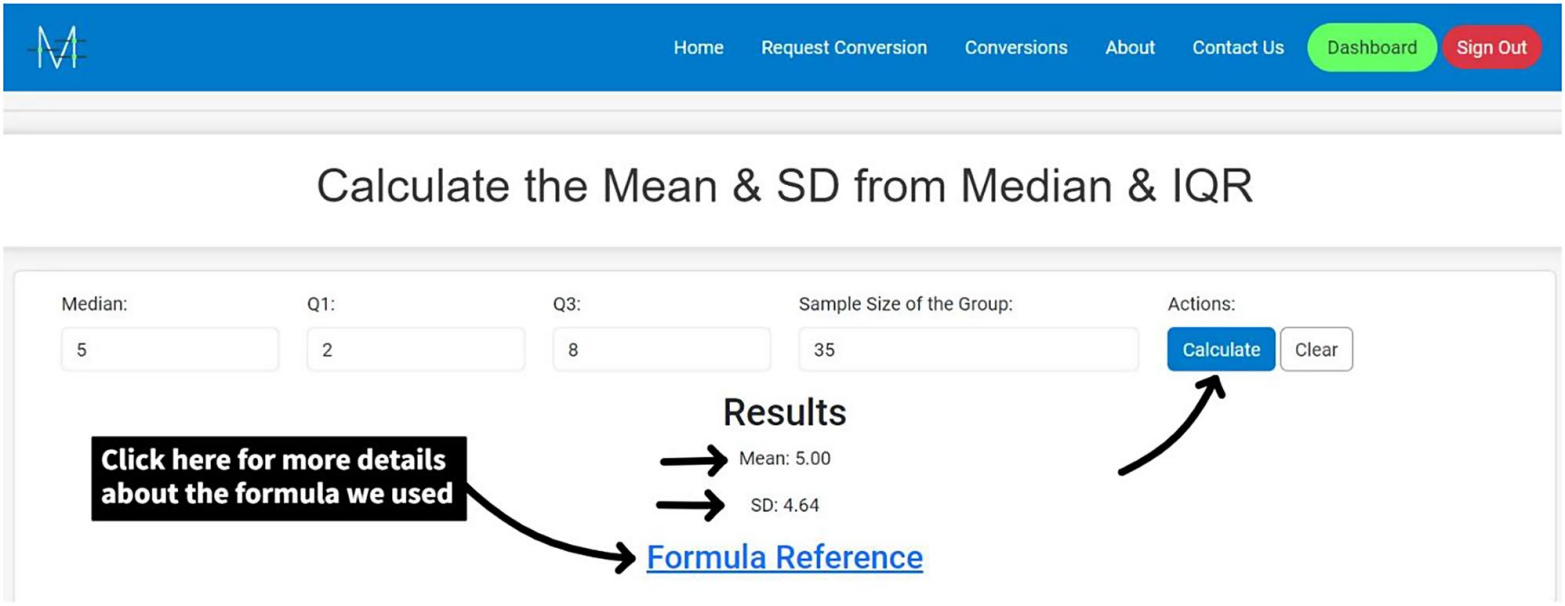
Click on Calculate to see the results

Using Meta-Analysis Accelerator is straightforward. Follow these steps to perform statistical data conversions:

1. **Access the Website**: Go to Meta-Analysis Accelerator (https://ma-accelerator.com/).
2. **Select the Conversions Page**: Click on the “Conversions” tab on the homepage to view the available conversions (Fig. 1).
3. **Choose the Appropriate Conversion:** Select the conversion that matches your needs (e.g., Median & interquartile range (IQR) to Mean & SD) (Fig. 2).
4. **Input Data**: Enter the required data in the provided fields. For example, for converting the “Median & IQR to Mean & SD”, input the median, Quartile 1 (Q1), Quartile 2 (Q2), and sample size (Fig. 3).
5. **Calculate**: Click the “Calculate” button to perform the conversion (Fig. 4).
6. **Review Results**: The tool will display the converted values, which you can copy and paste into your analysis software. Click “Clear” to enter new data or click the “Formula Reference” for more details about the calculation (Fig. 4).

By streamlining these conversions, Meta-Analysis Accelerator enhances the efficiency and accuracy of meta-analysis research, allowing researchers to focus on data interpretation and application.

### Development and design considerations

#### Software and hosting

Meta-Analysis Accelerator was developed using PHP [15] for backend processing. The user interface (UI) was designed using HTML [16], CSS [17], and JavaScript [18], with Bootstrap [19] providing responsive design elements. Figma [20] was used for User Interface/User Experience (UI/UX) design to ensure an intuitive and user-friendly experience. The tool is hosted on a cloud server provided by Hostinger [21], which ensures high availability and scalability. The hosting service provides robust security features to protect user data and maintain the tool’s integrity.

**Fig. 5:**
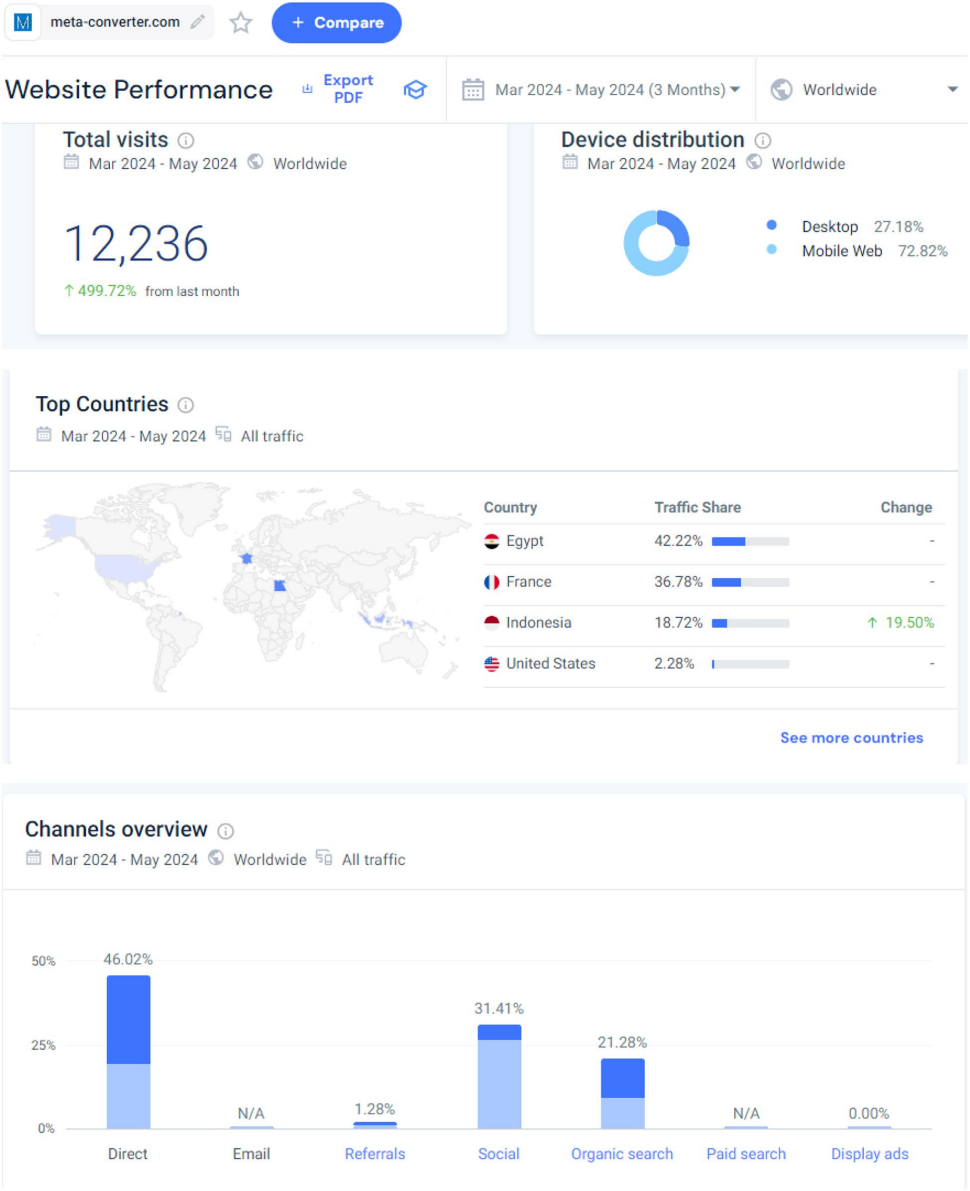
Statistics about the website usage

#### Design choices and iteration

Several design choices were driven by ease of use and familiarity. PHP was chosen for backend development due to its efficiency in web applications. Bootstrap was used for the UI because of its responsive design capabilities, which are crucial for a tool intended for global use. Figma was used for UI/UX design to create a seamless and visually appealing UI. The tool’s design went through multiple iterations based on user feedback. Initial designs focused on simplicity and ease of use, while later versions incorporated more advanced features and improvements suggested by users.

#### User testing and feedback

The tool underwent several rounds of internal testing by the development team to ensure functionality and accuracy. It was also tested by external users, including researchers and statisticians, to gather feedback and identify usability issues. User feedback plays a crucial role in guiding the development and enhancement of the platform, ensuring it meets the needs of the research community. The “Contact Us” page on the website allows users to submit their comments, suggestions, and issues directly, and this feedback is monitored regularly by the development team to identify areas for improvement and implement necessary changes. Additionally, user requests received via the “Request a Conversion” feature on the website have significantly influenced tool development, resulting in receiving six new conversion requests on top of the current 21 conversions since its launch.

## Results & discussion

The Meta-Analysis Accelerator was initially launched in October 2023 as Meta Converter (https://meta-converter.com/), later renamed to Meta-Analysis Accelerator (https://ma-accelerator.com/). This free tool was originally designed to meet a personal need but has since been widely adopted globally. From March 2024 to May 2024, it received 12,236 visits from various countries, including Egypt, France, Indonesia, and the USA, indicating its success and widespread use (Fig. 5). Approximately, 46% of the visits were direct, suggesting that users are intentionally accessing the tool, possibly indicating returning users or those who have bookmarked the tool due to its usefulness (Fig. 5). The website usage data was captured using SimilarWeb [22], integrated into Meta-Analysis Accelerator to monitor traffic and usage patterns.

Regarding data conversion, our platform employs validated statistical formulas, each supported by corresponding references. A single data conversion may utilize multiple statistical formulas, selected based on their prevalence and usability in research literature. While various formulas and data inputs can yield similar results, our future plans include enhancing user flexibility by allowing researchers to choose the specific formula for their data conversion needs. This approach ensures that researchers can tailor data processing according to their preferences, optimizing the platform’s utility and relevance in supporting systematic reviews and meta-analyses.

Currently, Meta-Analysis Accelerator offers 21 conversions, each requiring specific data inputs (Table 1).

**Table 1:** Conversions available in meta-analysis accelerator with the required inputs

Conversion Method	Inputs Required	Calculation Description
Change in Mean and SD from Baseline [23]	Means before and after, Correlation coefficient (CC)	Calculate mean change and SD change accounting for within-person correlation.
SD to CC [24]	Baseline SD, Post-treatment SD, SD change	Compute CC based on provided SDs.
IQR to Mean & SD [25]	Median, Q1, Q3, Sample size	Derive mean and SD using Wan et al. method from IQR data.
Standard Error of the Mean (SEM) to SD [26]	SEM, Sample size	Convert SEM to SD for better estimation of data dispersion.
Confidence interval (CI) to SD in One Group [26]	CI level, Number of participants, Upper and Lower limits	Estimate SD from CI data to interpret result significance.
Median & Range to Mean & SD [25]	Median, Minimum, Maximum, Sample size	Calculate mean and SD using Wan et al. method from median and range data.
Range to SD [27]	Minimum, Maximum, Sample size	Provide estimated SD from range data.
Means & SDs to Cohen’s d and Effect Size Correlation (r) [28]	Means and SDs of two groups	Compute Cohen’s d and r for group comparison.
t-value & Degrees of Freedom to Cohen’s d [28]	t-value, Degrees of freedom	Derive Cohen’s d and r from t-test results.
P-value to SD in Two Groups [29]	P-value, Group sizes, Means	Estimate SD based on group comparison p-value for result interpretation.
Combining Means & SDs from Multiple Groups [30]	Means, SDs, Totals of multiple groups	Aggregate data into unified mean and SD values for comprehensive analysis.
CI Limits & Participants to SD [29]	Upper and Lower CI limits, CI level, Group size	Calculate SD from CI data to interpret data precision.
List of Values to Statistics [31]	List of patient values	Derive sample size, mean, SD, variance, sum, SEM, and margin of error from input data list.
OR to Cohen’s d [32]	OR value	Convert OR to Cohen’s d effect size for OR interpretation.
Unadjusted OR Calculator [30]	Event counts, Totals for two groups	Compute OR with CI, Z-score, and p-value for quick OR analysis.
Unadjusted RR Calculator [30]	Event counts, Totals for two groups	Calculate RR with CI, Z-score, and p-value for quick RR analysis.
Unadjusted Risk Difference (RD) Calculator [30]	Event counts, Totals for each group	Determine RD with CI, Z-score, and p-value for quick RD analysis.
Measurement Units Converter [33]	Measurement value, Units	Convert between g/L, µg/L, mg/dL for accurate measurement unit transformation.
Time Units Converter [34]	Time value, Units	Convert time intervals between hours, days, months, and years for easy time unit adjustment.
Lipids Converter [35]	Lipid measurement value, Units	Convert between mmol/L and mg/dL for various lipid types such as HDL, LDL, VLDL, Triglycerides, Cholesterol.
Glucose Converter [36]	Glucose measurement value, Units	Convert between mmol/L and mg/dL for accurate glucose measurement transformation.

**Fig. 6:**
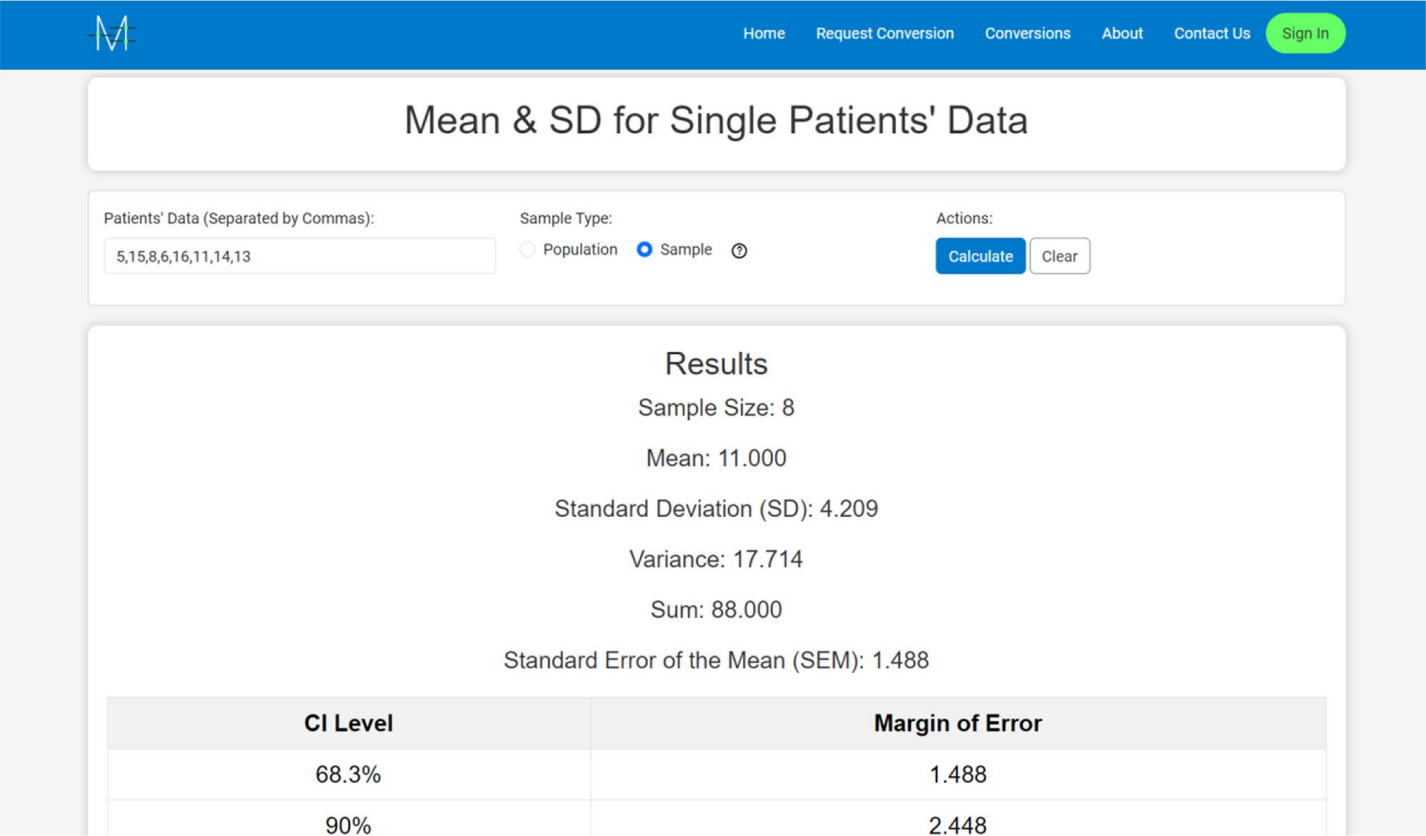
Calculate the mean and SD for single patients’ data

Before the launch of Meta-Analysis Accelerator, there was no online software that included most of the statistical conversions used in meta-analysis. The traditional method involved using Microsoft Excel sheets [37], which were shareable among researchers and prepared by statisticians for the data extraction phase. However, this method has several limitations. Firstly, there is an issue with cross-device accessibility; Microsoft Excel is not accessible on mobile phones and is difficult to use on tablets. Secondly, it is very expensive. Thirdly, it is not always functional and could hardly be used in some scenarios. For example, if I have data for each patient regarding a variable like age, I will have to calculate the overall mean and SD. While this is possible in Microsoft Excel using functions like AVERAGE and STDEV.P/STDEV.S, Meta-Analysis Accelerator makes the process more straightforward and user-friendly by allowing the data to be input directly and calculating the sample size, mean, SD, and margins of errors with a single click (Fig. 6).

**Fig. 7:**
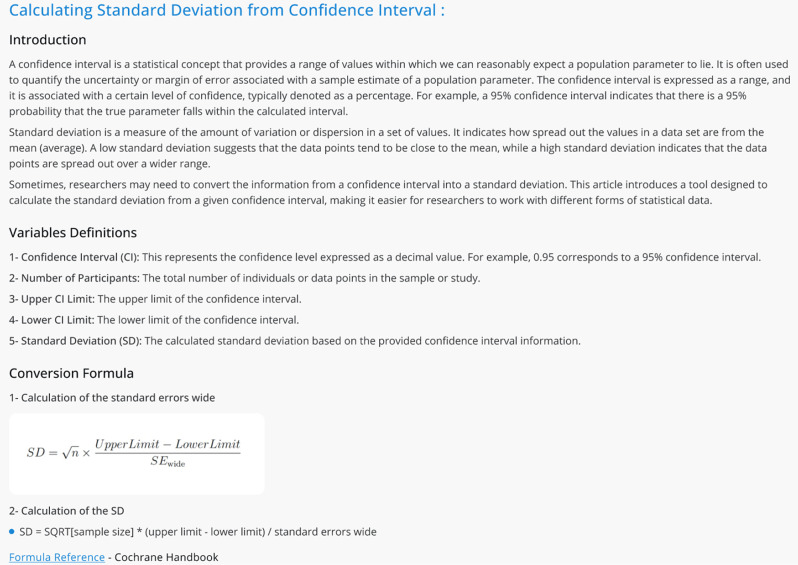
Example for the articles that will be added

**Fig. 8:**
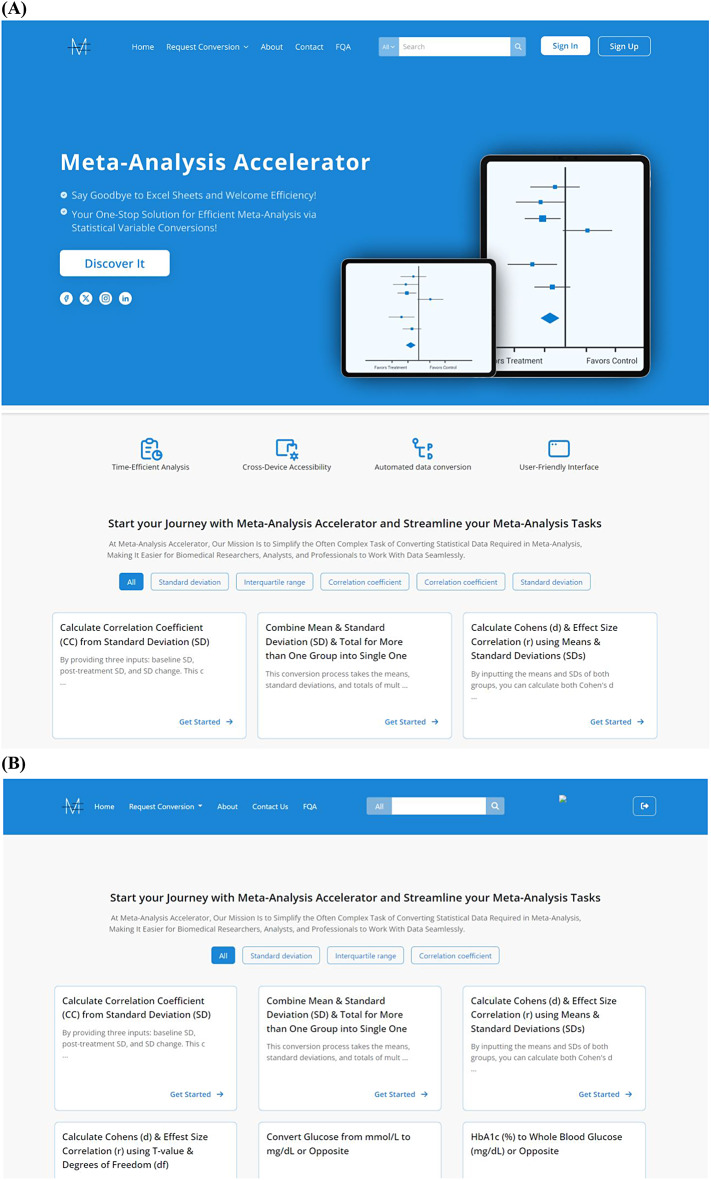
Screenshots from the new upcoming design: **(A)** Home page, **(B)** Conversion page

## Limitations and future work

1. **Manual Data Input**: Currently, the tool requires manual data input, which can be time-consuming for large datasets. Future updates will include the ability to upload data files (e.g., .csv) for batch processing.
2. **Export Option**: We acknowledge that manually copying and pasting results can be cumbersome. To streamline data handling, we plan to introduce an export feature, allowing users to download converted data in formats compatible with popular analysis tools, such as RevMan, as well as in spreadsheet formats like Excel.
3. **Limitations of Statistical Conversions**: While Meta-Analysis Accelerator provides a convenient platform for performing various statistical conversions, it is crucial for users to understand the limitations and assumptions associated with these conversions. For instance, converting medians and IQRs to means and SDs should only be done under specific circumstances and with strong assumptions about the data distribution.
4. **Warning Signs and Pop-up Notifications**: To address potential misuse, we plan to implement warning signs and pop-up notifications within the tool. These warnings will prompt users to verify the appropriateness of their intended conversions before proceeding. This feature will help users understand the methodological and clinical heterogeneity of their data and ensure that they are making valid conversions.
5. **User Awareness and Education**: Users are encouraged to carefully consider the assumptions behind each conversion and consult relevant statistical guidelines to determine the appropriateness of their analyses. By including these warnings, we aim to enhance user awareness and prevent potential misuse of the tool. Additionally, the explanatory articles accompanying each conversion will further raise awareness and provide education on the variables and formulas involved, helping users better understand the methodologies and make informed decisions in their analyses.
6. **Upcoming Features**:
  In response to user requests, we are working on incorporating six new conversions in the near future:
    1. To convert the HR and CI to log HR and standard error (SE) of the log HR.
    2. To calculate the pre-mean and SD from the post-mean and SD and change in mean and SD.
    3. SD to SEM.
    4. Least Squares mean to mean.
    5. Mean and SD to median and IQR.
    6. HR to OR.
  Each conversion will be accompanied by an explanatory article detailing the variables and formulas involved (Fig. 7). We hope these articles will raise awareness and provide education about the formulas used, thereby reducing the potential for misuse of the tool.
  We will utilize UI/UX to improve user experience and ensure the website is responsive and optimized for various devices (Fig. 8).
  Tags will be added in addition to the current search bar to facilitate quick access to the conversion tools (Fig. 8).
  A **community feature** will be added to the website, allowing users to connect, follow each other, and ask questions related to meta-analysis.
  Since the same data conversion can be done depending on more than one statistical formula with a very similar result, we also plan to give the user the option to choose the statistical formula he prefers to conduct the data conversion.
  The input and output data from the platform will be made via a spreadsheet form to facilitate data entry. The final spreadsheet file product will have all the estimates and conversions in one place, reducing data entry errors and allowing researchers to check and revise all conversions simultaneously.

## Conclusions

Meta-Analysis Accelerator represents a significant advancement in the field of systematic reviews and meta-analysis, addressing the critical pain points researchers face with statistical data conversion. By offering a comprehensive variety of conversions in a user-friendly platform, it ensures accuracy, efficiency, and consistency in data transformation. With continuous improvements and planned new features, Meta-Analysis Accelerator is poised to remain an indispensable resource for researchers, enhancing the quality and ease of conducting meta-analyses.

## Availability and requirements

- **Project Name**: Meta-Analysis Accelerator.
- **Project Home Page**: https://ma-accelerator.com.
- **Operating System(s)**: Platform independent.
- **Programming Language**: HTML, CSS, JavaScript, Bootstrap, PHP, and Laravel.
- **License**: Free license.
- **Restrictions to Use by Non-Academics**: None.

## Data Availability

No datasets were generated or analysed during the current study.

## Abbreviations

CI: Confidence Interval
RD: Risk Difference
IQR: Interquartile Range
r: Effect Size Correlation Coefficient
SD: Standard Deviation
SEM: Standard Error of the Mean
UI/UX: User Interface/ User Experience
OR: Odds Ratio
RR: Risk Ratio
HR: Hazard Ratio
SEM: Standard Error of the Mean
g/L: Grams per Liter
µg/L: Micrograms per Liter
mg/dL: Milligrams per Deciliter
mmol/L: Millimoles per Liter
HDL: High-Density Lipoprotein
LDL: Low-Density Lipoprotein
SE: Standard Error
CC: Correlation Coefficient
